# Effects of conventional immunosuppressive treatment on CD244+ (CD28null) and FOXP3+ T cells in the inflamed muscle of patients with polymyositis and dermatomyositis

**DOI:** 10.1186/s13075-016-0974-5

**Published:** 2016-04-01

**Authors:** Jayesh M. Pandya, Ingela Loell, Mohammad Shahadat Hossain, Mei Zong, Helene Alexanderson, Sukanya Raghavan, Ingrid E. Lundberg, Vivianne Malmström

**Affiliations:** Rheumatology Unit, Department of Medicine, Center for Molecular Medicine, Karolinska University Hospital, Solna, Karolinska Institutet, Stockholm, Sweden; Department of NVS, Division of Physiotherapy, Solna, Karolinska Institutet, Huddinge and Physiotherapy Clinic, Karolinska University Hospital, Stockholm, Sweden; Present address: Department of Microbiology and Immunology, Institute for Biomedicine, Gothenburg University, Gothenburg, Sweden

**Keywords:** T-lymphocyte, Myositis, Treg cells, Glucocorticoids, Inflammation

## Abstract

**Background:**

T-cell infiltrates may persist in muscle tissue of polymyositis (PM) and dermatomyositis (DM) patients despite aggressive immunosuppressive treatment. Here, we investigated to what extent persistent T cells in affected muscle were FOXP3+, a marker for regulatory T cells (Tregs), or CD244+, a marker for CD28null T cells, and whether their presence correlated to clinical outcome. The sensitivity of CD28null T cells towards glucocorticoid and Treg-mediated immunosuppression was also investigated.

**Methods:**

Muscle biopsies from 16 newly diagnosed or untreated patients with PM/DM were investigated by immunohistochemistry for expression of CD3, FOXP3 and CD244 before and after treatment with glucocorticoids and immunosuppressive agents. For clinical evaluation, serum levels of creatine kinase, muscle performance (FI and MMT8), disease activity (MITAX) and disability (HAQ) were measured. In vitro suppressive effects of glucocorticoids and Tregs on T-cell activation were measured by CD69 upregulation.

**Results:**

Before treatment, CD244+ cells were present at higher proportions compared to FOXP3+ cells in the inflamed muscle. Following treatment, FOXP3+ cell numbers decreased while CD244+ cells persisted. Patients with impaired muscle function (<75 % FI) post-treatment had higher levels of CD244+ cells in the follow-up biopsy compared to those with FI >75 %. MITAX and HAQ correlated with the number of CD244+ cells post-treatment. CD4+CD28null T cells displayed lower sensitivity towards both glucocorticoid and Treg-mediated immunosuppression in vitro compared to their CD28+ counterparts.

**Conclusions:**

Poor outcome in patients with myositis following immunosuppressive therapy was linked to persistence of CD244+ (CD28null) T cells in muscle tissue, suggesting their resistance against immunosuppression. A relative loss of regulatory T cells could also contribute to poor clinical outcome given their recently ascribed role in muscle tissue regeneration.

## Background

Polymyositis (PM) and dermatomyositis (DM) are characterized by chronic muscle weakness and inflammation in muscle tissue leading to disability, decreased quality of life and reduced life expectancy. Histopathologically, these myopathies are characterized by immune cell infiltrates, mainly T cells and macrophages in the skeletal muscle tissue [1–3].

Conventional treatment of PM and DM is based on the use of glucocorticoids in high doses over an extended period of time together with additional immunosuppressive agents [4]. More recently, exercise has also become an important part of the treatment [5]. However, the treatment outcome is unpredictable in the patients [4]. In some patients, the inflammatory infiltrate in muscle tissue persists despite aggressive immunosuppressive treatment and is associated with remaining muscle weakness [6–8]. In this context, the CD28null T cells are of particular interest as they are long-lived and suggested to be resistant to apoptosis [9–12]. CD28null T cells are highly differentiated cells lacking the co-stimulatory molecule CD28, are often clonally expanded and display proinflammatory effector functions such as interferon gamma (IFNγ) and tumor necrosis factor (TNF) production as well as cytotoxic capacity and upregulation of activating receptors mostly associated with natural killer (NK) cells [13–15]. Frequencies of CD28null T-cell subsets are higher in CD8 as compared to CD4 lineage, but still relatively low in healthy individuals [16] but are increased in the elderly [17] and in various chronic inflammatory and autoimmune conditions [14, 18–22]. Contrary to these proinflammatory cells, FOXP3+ regulatory T cells (Tregs) are key players in the maintenance of peripheral tolerance by limiting T-cell activation and effector function [23, 24]. Interestingly, there is a growing body of data indicating that tissue-resident FOXP3+ Tregs are also instrumental for repair and tissue regeneration, and for muscle this can be accomplished by both direct effects on muscle precursor cells [25] and via the growth factor amphiregulin [26]. No data in this context are so far available for patients with myositis.

Recent results from our group demonstrate that CD244 can be used as a surrogate marker to identify CD28null T cells in the circulation and in the muscle tissue of myositis patients, and also that the majority of the muscle-infiltrating T cells in myositis patients are of the proinflammatory CD28null phenotype [27, 28]. However, FOXP3+ Tregs have also been described in myositis muscle tissue [29]. Interestingly, it has been demonstrated in peripheral blood mononuclear cells (PBMCs) from healthy donors that CD28null T-cell proliferation and function could only partly be suppressed by Tregs [30]. This led to our interest towards the efficacy of Tregs and glucocorticoids in limiting local CD28null T-cell activation and persistence in the setting of myositis.

In the present study we have investigated the effects of immunosuppressive treatment on CD3+ (T-cell marker), CD244+ (CD28null T-cell marker) and FOXP3+ (regulatory T-cell marker) cells in myositis muscle tissue, and its relation to the short- and long-term clinical outcomes for patients with PM and DM, as well as the in vitro immunosuppressive effects on circulating CD28null T cells. We demonstrate here that a poor outcome following glucocorticoid therapy is linked to persistence of CD244+ cells in muscle tissue, i.e., CD28null T cells and a relative decrease of Tregs following such therapy. Furthermore, CD28null T cells isolated from the circulation of myositis patients were resistant to both glucocorticoid and Treg-mediated immunosuppression in vitro.

## Methods

### Patients, treatment and healthy donors

This is an observational, prospective study including newly diagnosed patients at the Karolinska University Hospital with definite or probable PM or DM who gave consent to have a re-biopsy after treatment with conventional immunosuppressive treatment [31, 32]. A second biopsy was planned after 6 months with immunosuppressive treatment including prednisolone with a starting dose of 40–60 mg/day, slowly tapering, in combination with a disease-modifying drug according to the treating physician’s choice (azathioprine 2 mg/kg/day or methotrexate 15–20 mg/week) (Table 1). Muscle biopsies taken before and after treatment were available from sixteen patients (Table 1). Only those who had at least 10 CD3+ cells in muscle-biopsy sections with a minimum of 2 CD3+ cells/mm^2^ either before or after treatment (*n* = 14) (patients 1–14; Table 1) were included for T cell phenotype analysis in muscle tissue and clinical evaluation post-treatment. For long-term clinical follow-up, all sixteen patients were included. The patients were followed with registration of clinical data in the SweMyoNet registry. Data from a time point 5 to 10 years from start of treatment were included to determine long-term outcomes.

**Table 1:** Patient demography and treatment

Patient no.	Diagnosis	Treatment duration at second biopsy (months)	Cumulative prednisolone dose at second biopsy (mg)	Other immunosuppressive agents at second biopsy	ANA (+/neg) and pattern	Autoantibodies detected in line immunoassay
1	DM	8	6005	MTX	+, granular	neg
2	DM	5	5210	AZA	+, granular	neg
3	DM	7	7875	None	+, granular	neg
4	DM^a^	10	4778	MTX	+, granular	anti-Ro-52, anti-Ro-60, anti-LA
5	DM	11	7179	AZA	+, granular	neg
6	DM	9	8668	AZA	neg	anti-Ro-52
7	DM^b^	4	3935	None	+, granular	anti-Ro-52, anti-PL7
8	DM^b^	8	NA	AZA, CsA	+, granular	anti-Ro-52
9	PM	6	5250	MTX	+, granular and homogenous	anti-Ro-52, anti-SRP
10	PM	12	9090	AZA	+, granular	neg
11	PM	11	9013	MTX, AZA	NA	NA
12	PM^b^	16	6775	AZA	neg	anti-Mi2
13	PM^b^	4	5525	CYC	neg	anti-Jo-1, anti-Ro-52
14	PM^b^	6	1360	None	neg	neg
15	PM	9	3702	AZA	neg	anti-Ro-52, anti-Ro-60
16	PM	8	8435	AZA	+, granular	anti-Ro-52

^a^Additional diagnosis Sjögren's syndrome.

^b^Diagnosis probable

+ positive, *ANA* anti-nuclear antibodies, *AZA* azathioprine, *CsA* cyclosporine A, *CYC* cyclophosphamide, *DM* dermatomyositis, *MTX* methotrexate, *NA* not available, *neg* negative, *PM* polymyositis

For in vitro immunosuppression assays, PBMCs from 6 untreated myositis patients (2 DM, 4 PM; median age 63.5 (43–74) years) and 6 healthy donors (buffy coats), all with at least 2 % CD4+ CD28null T-cell frequency (patients, median 15.2 %, range 2.01–22.8 %; healthy donors, median 5.9 %, range 2.08–14.6 %) in peripheral blood were obtained.

### Ethics, consent and permissions

All participants gave informed consent to participate in the study, which was approved by the regional Human Ethics Committee at Karolinska Institutet, Stockholm.

### Autoantibodies (as listed in Table 1)

Patient sera were tested for antinuclear antibodies (ANA) by indirect immunofluorescence as a routine test using Hep-2 KS cells and fluorescein-labeled anti-human IgG at the Department of Clinical Immunology, Karolinska University Hospital. Myositis-specific and -associated autoantibodies were identified by line immunoassay (Myositis Profile Euroline, Euroimmun, Lubeck, Germany) by Dr. P. Charles, Kennedy Institute of Rheumatology, London, UK [33].

### Muscle biopsy specimens and immunohistochemistry analysis

Biopsy specimens were obtained from the vastus-lateralis or tibialis-anterior muscle by a “semi-open” technique under local anesthesia [34, 35], before and after treatment. The muscle biopsies were immediately frozen in isopentane, chilled by liquid nitrogen, stored at –70 °C, and 7-μm thick biopsy sections were prepared for immunohistochemistry. As demonstrated previously by Fasth et al., CD244 was used as a surrogate marker to detect the presence of CD28null T cells in muscle tissue of DM and PM patients [27]. This facilitates direct quantification of CD28null T cells (which are highly differentiated effector T cells) and reduces the risk for inclusion of recently activated T cells temporarily downregulating CD28. Therefore, in order to quantify the total number of T cells in muscle tissue and the fraction of CD244+ T cells, serial sections of patient muscle biopsies were stained for CD3 and CD244 using immunohistochemistry. To quantify the number of Tregs, muscle biopsy sections were stained for FOXP3. Mouse monoclonal anti-human CD3 (clone SK7; Becton Dickinson, USA), goat anti-human CD244 (R&D Systems, Minneapolis, MN, USA) and mouse anti-human Foxp3 (IgG1, clone 236/E7, 1; eBioscience, San Diego, CA, USA) antibodies were used to detect the presence of CD3, CD244 and FOXP3, respectively. Respective isotype control antibodies were irrelevant mouse IgG1 (DAKO, Glostrup, Denmark) or goat IgG (Caltag Laboratories). Stainings were performed as described elsewhere [28, 36].

Stained tissue sections were examined using a Polyvar II microscope (Reichert-Jung, Vienna, Austria) and a Leica DM RXA2 microscope (Leica Microsystems, Wetzlar, Germany) and photographed with a Leica DC digital color video camera 300 F (Leica Microsystems DI, Cambridge, UK). The number of cells expressing CD244, FOXP3 and CD3 per unit area (mm^2^) was assessed quantitatively using computer-assisted image analysis. Prior to the microscopic evaluation, slides were coded by a third person and analysis was blinded.

### Clinical outcome measures

For clinical evaluation, post-treatment muscle performance was measured by the disease-specific Functional Index (FI) of myositis at biopsy time points [37]. Post-treatment 5-year follow-up of disease activity was performed by the Myositis Intention To Treat Activity Index (MITAX) [38] and muscle strength was measured by Manual Muscle Testing 8 (MMT8) [39]. Additionally, to measure limitations in daily activities and disability at 5-year and 6- to 10-year follow-up, the Health Assessment Questionnaire (HAQ) disability index was employed [40]. Detailed information about MITAX, MMT8 and HAQ can be found on the "Disease Activity Core Set Measures" section of the International Myositis Assessment and Clinical Studies Group (IMACS) webpage. (http://www.niehs.nih.gov/research/resources/imacs/index.cfm). Serum levels of creatine kinase (s-CK) at the time of muscle biopsy were analyzed as a measurement of muscle damage as routine analyses at the Department of Clinical Chemistry at Karolinska University Hospital.

### In vitro immunosuppression assay

The immunosuppressive potential of Tregs on T-cell subsets was measured using BD FastImmune™ Human Regulatory T Cell Function Kit (BD Bioscience, USA), with a slightly modified protocol. Glucocorticoid-mediated suppression on T-cell subsets was also studied. For in vitro assays, RPMI + 2 mM L-glutamin + 100 units/ml penicillin + 100 μg/ml streptomycin + 10 μM Hepes (all from Sigma-Aldrich) + 10 % pooled human AB serum (from the Karolinska University Hospital Blood Center) was used as the cell culture medium. PBMCs were stimulated with plate-bound anti-CD3 (2.5 μg/ml, OKT-3) in U-bottomed 96-well plates. At the same time, either glucocorticoid (2 μg/ml, Solu-Medrol®, Pfizer) or Tregs (Tregs to target cell/PBMC ratio 1:8) were added. Tregs were isolated from PBMCs using immunostaining with the following anti-human antibodies: CD4-FITC, CD3-PerCp-Cy5.5 and CD25-PE (BD Bioscience). Tregs were sorted on a MoFlow high-speed cell sorter (Beckman Coulter, USA) as CD3+CD4+CD25^high^. The cell cultures were kept for 7–10 h at 37 °C, 5 % CO_2_, and stained with the following anti-human antibodies: CD4-FITC, CD25-PE, CD3-PerCP-Cy5.5; CD69-PE-Cy7; CD28-APC (BD Bioscience). Stained cells were acquired by flow cytometry on a CyAn (Beckman Coulter, USA) and the data were analyzed by FlowJo software version 9.0 (Tree star Inc. Ashland, Oregon, USA). T-cell activation, as measured by CD69 expression, was analyzed on the effector T-cell subsets divided into CD4+CD28+ or CD4+CD28null T cells based on CD28 expression. Suppression, by either glucocorticoid or Tregs, was quantified as percentage reduction in upregulation of the activation marker CD69 by measuring geometrical mean fluorescence intensity (GMFI) of samples compared to stimulated (STM) and unstimulated (UNSTM) controls using the following formula: % suppression = (GMFI of STM – GMFI of sample) × 100/(GMFI of STM – GMFI of UNSTM).

### Statistical analysis and graphing

Correlations in the study were analyzed using the two-tailed Spearman correlation test. The Spearman correlation coefficient (r_s_) was interpreted as follow for the degree of correlation: 0–0.25 as no or very low, 0.26–0.40 as low, 0.41–0.69 as moderate, 0.70–0.89 as high, and 0.90–1.0 as very high correlation [41]. The Mann-Whitney two-tailed test was used to compare CD3+ cells/mm^2^ and CD244+ cells/mm^2^ between ΔFI groups and FI groups post-treatment (FI-high and FI-low groups). For all paired comparisons, two-tailed Wilcoxon matched-pairs signed rank test was used. The software program GraphPad Prism (version 5.0 for Mac) was used for all graphing and statistical analysis including linear regression analysis.

## Results

### Despite a good clinical response at the group level, many patients regained less than 75 % of their muscle function

**Fig. 1:**
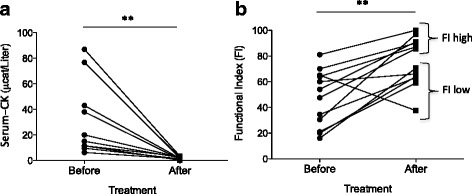
Clinical evaluation of patients before and after immunosuppressive treatment. Serum creatine kinase (*CK*) levels decreased for all patients post-treatment (**a**). At the group level, Functional Index (*FI*) increased from 50.7 % (median) to 78.1 % post-treatment (*n* = 12) (**b**). Based on FI score post-treatment, patients clustered into two groups: one group with FI higher than 75 % (FI-high group, *n* = 6) and another with FI less than 75 % (FI-low group, *n* = 6) (**b**)

For the cohort of 16 patients (11 females, 5 males), the median age at the time of first biopsy was 61.5 (range 41–88) years (Table 1). At the time of the repeat biopsy, median treatment duration was 8 months (range 4–16), with a median cumulative prednisolone dose of 6005 mg (1360–9090 mg). Overall, the patient cohort responded well to treatment. Out of the 14 patients included in the T-cell phenotyping, s-CK data were available from 11 patients and FI data were available from 12 patients. s-CK levels had decreased at follow-up, i.e., time of the second biopsy (median before 20.0 μcat/L, after 1.9 μcat/L; *p* = 0.003) (Fig. 1a). Improvement in FI measurement was displayed in 11 out of 12 patients. At the group level, FI improved from 50.7 % (median) to 78.1 % post-treatment (*p* = 0.005) (Fig. 1b). Eight out of 12 patients displayed more than 20 % improvement in the FI (i.e., ΔFI).

Based on the levels of FI score post-treatment, patients clustered into two groups: one group with FI higher than 75 % (FI-high group, range 85.8–100, *n* = 6) and another with FI less than 75 % (FI-low group, range 37.5–70.5, *n* = 6), and hence less good clinical response (Fig. 1b).

### CD244+ cells dominate the T-cell infiltrate in inflamed myositis muscle tissue

**Fig. 2:**
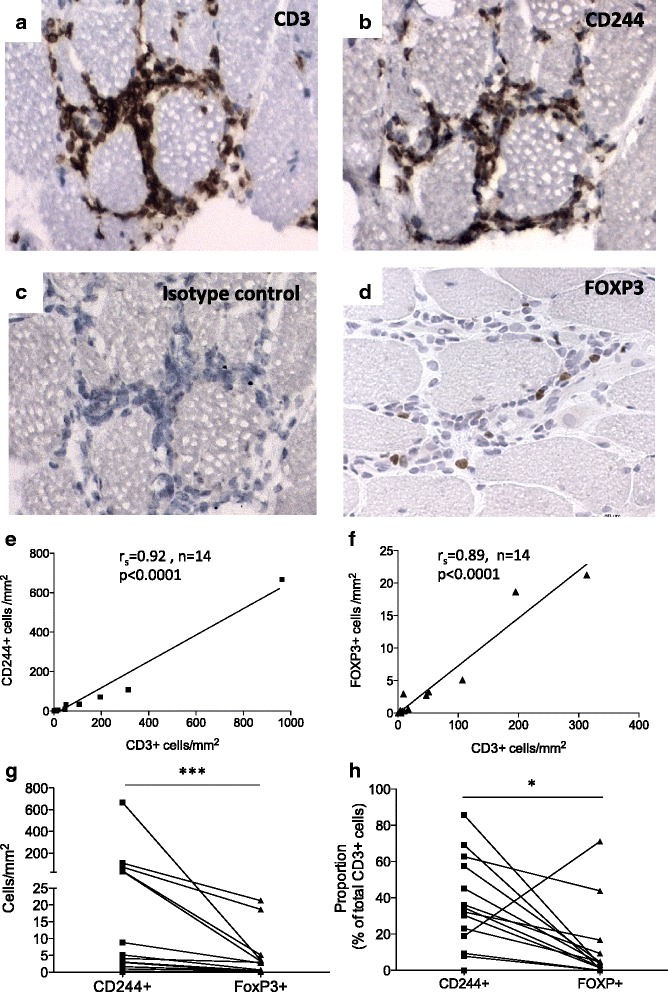
Expression of CD244 (CD28null) and FOXP3 among T-cell infiltrates in muscle tissue of myositis patients. Immunohistochemistry of muscle tissue obtained from the patients before the start of immunosuppressive treatment are shown with representative staining of CD3 (**a**), CD244 (**b**), isotype control for CD244 (**c**) and FOXP3 (**d**). Both CD244+ cells/mm^2^ (95 % CI = 0.75 to 0.97) (**e**) and FOXP3+ cells/mm^2^ (95 % CI = 0.66 to 0.96) (**f**) strongly correlated with CD3+ cells/mm^2^. The number of FOXP3+ cells was significantly lower than CD244+ cells in myositis muscle tissue (**g**). When compared to total CD3+ cells, CD244+ cells were present in approximately 10-fold higher proportion compared with FOXP3+ in the myositis muscle tissue (**h**). *p < 0.05, ***p < 0.001. *CI* confidence interval, *r*_*s*_ Spearman correlation coefficient

Muscle biopsy sections were utilized to study CD3+, CD244+ and FOXP3+ cells in myositis muscle tissue. Figure 2a–c depicts representative examples of CD3, CD244 and isotype control staining. Figure 2d is a representative staining for FOXP3 in muscle tissue of myositis patients. Of the 14 patients analyzed, the number of CD3+ T cells before the start of treatment ranged between 0.5–963 cells/mm^2^ (median 13.5) (Fig. 2e) while the number of CD244+ cells varied between 0–666 cells/mm^2^ (median 4.6) (Fig. 2e). Most areas positive for CD3 also stained positive for CD244 on consecutive sections (Fig. 2a and b). Both CD244+ cells/mm^2^ (r_s_ = 0.92, *p* < 0.0001) and FOXP3+ cells/mm^2^ (r_s_ = 0.89, *p* < 0.0001) correlated strongly with CD3+ cells/mm^2^, strengthening the data that CD244 and FOXP3 staining is T-cell specific (Fig. 2e and f). Note that even after removing the outlier in Fig. 2e, a strong correlation was observed (r_s_ = 0.90, *p* < 0.0001).

FOXP3+ cells were present in lower numbers than CD244+ cells in myositis muscle tissue (median 1.69 FOXP3+ cells/mm^2^, range 0–21.3, *p* = 0.0002) (Fig. 2g). When comparing the proportions (% of total T cells), CD244+ cells (median 33.4 %, range 0–85.7 %) were present in approximately 10-fold higher proportion compared to FOXP3+ cells (median 3.4 %, range 0–71.1 %, *p* = 0.01) in the myositis muscle tissue (Fig. 2h).

### CD244+ cells persist post-treatment in myositis muscle tissue, while the number of FOXP3+ cells decreases

**Fig. 3:**
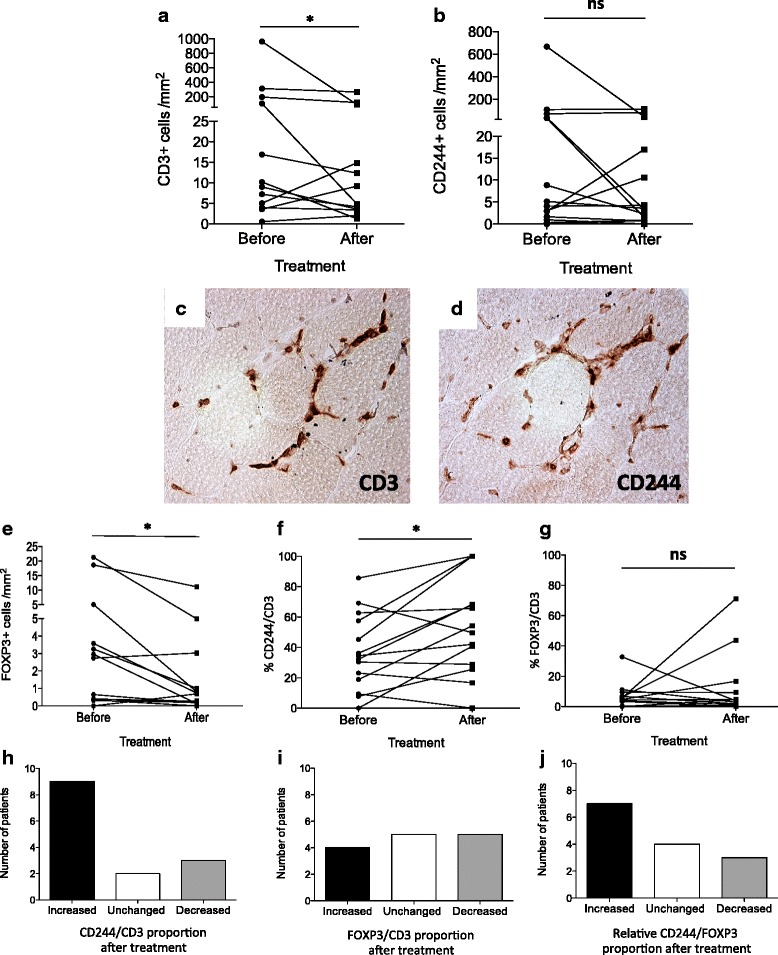
Effects of immunosuppressive treatment on CD244 (CD28null) and FOXP3 expressing cells in muscle tissue. Lower number of T cells (CD3+ cells) was seen in post-treatment muscle tissue compare to muscle tissue obtained before treatment (**a**) while the number of CD244+ cell/mm^2^ did not change significantly post-treatment (**b**). Representative staining displaying remaining CD3+ cells in muscle tissue obtained post-treatment (**c**) and persistence of CD244+ cells among CD3+ T cells in the consecutive muscle biopsy section are shown (**d**). The number of FOXP3+ cells was lower post-treatment in comparison with before treatment (**e**). The CD244/CD3 proportion increased in post-treatment muscle tissue (**f**) while the FOXP3/CD3 proportion did not change significantly (**g**). At the individual patient level, a majority (9/14) of patients displayed increases in CD244/CD3 proportion post-treatment (**h**) while the FOXP3/CD3 proportion was unchanged or lower for the majority of patients (10/14) (**i**). The relative proportion of CD244/FOXP3 increased for 7, remained unchanged for 4 and decreased for 3 patients post-treatment (**j**). *p < 0.05. *ns* non-significant

After a median 8 months of treatment, the median T-cell number in muscle tissue was 4.5 cells/mm^2^, which was significantly lower compared to before treatment (13.5 cells/mm^2^, *p* = 0.02) (Fig. 3a). However, the number of CD244+ cells/mm^2^ did not change significantly post-treatment (*p* = 0.58) (Fig. 3b). Representative staining displaying persistence of CD244+ cells among CD3+ cells in post-treatment muscle biopsies is shown in Fig. 3c and d. On the contrary, the number of FOXP3+ cells was lower, 0.48 cells/mm^2^ (median) compared to before treatment 1.69 cells/mm^2^ (*p* = 0.01) (Fig. 3e).

The persistence of CD244+ cells led to an increase in CD244+/CD3+ proportion from 33.3 % (median) to 51.9 % in post-treatment muscle tissue (*p* = 0.02) (Fig. 3f), while the FOXP3+/CD3+ proportion did not change (*p* = 0.79) (Fig. 3g). Also at the individual patient level, a majority (9/14) of patients displayed increases in CD244+/CD3+ proportion post-treatment (Fig. 3h), while the FOXP3+/CD3+ proportion was unchanged or lower for the majority of patients (10/14) (Fig. 3i). The relative proportion of CD244/FOXP3 increased for 7 patients, remained unchanged for 4 patients and decreased for 3 patients post-treatment (Fig. 3j). These results indicate that treatment led to a relative decrease in FOXP3/CD3 proportion compared to the CD244/CD3 proportion for the majority of patients.

### Persistence of CD244+ cells in muscle tissue is linked to relatively poor outcome following glucocorticoid therapy

**Fig. 4:**
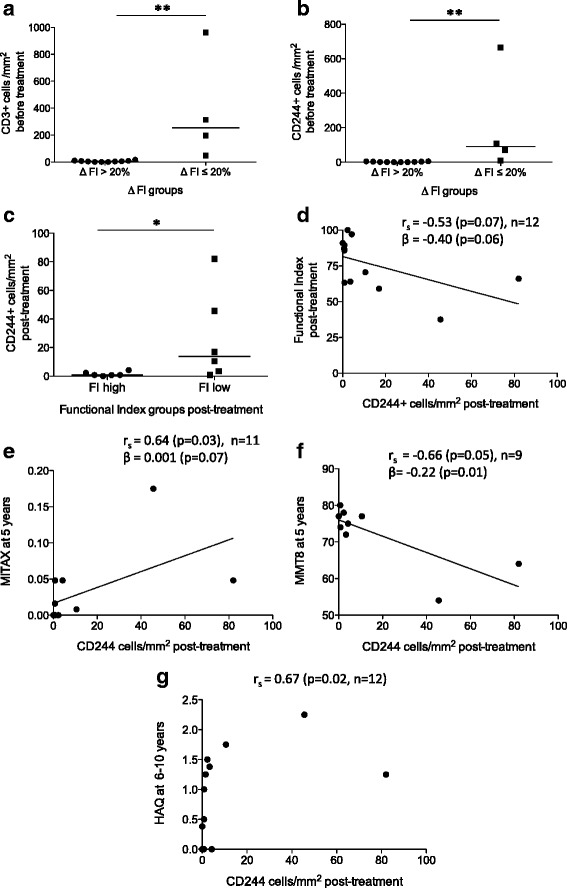
Correlation of the clinical response with the persistence of CD244+ cells in muscle tissue. Patients with low Functional Index (*FI*) improvement (ΔFI ≤20 %, *n* = 4) had significantly higher number of CD3+ cells/mm^2^ (**a**) and CD244+ cells/mm^2^ (**b**) before treatment, compared to the patient group with high FI improvement (ΔFI >20 %, *n* = 8). Patients in the FI-low group (FI <75 %) post-treatment had higher levels of CD244+ (CD28null) T cells in post-treatment muscle biopsies compared to those in the FI-high group (FI >75 %) (**c**). CD244+/mm^2^ in post-treatment muscle biopsies displayed negative correlation with FI after treatment (95 % CI = –0.85 to 0.08) (**d**). CD244+ cells/mm^2^ post-treatment correlated significantly with Myositis Intention To Treat Activity Index (*MITAX*) at 5-year follow-up (95 % CI = 0.05 to 0.90) (**e**). CD244+ cells/mm^2^ post-treatment displayed negative correlation with Manual Muscle Testing 8 (*MMT8*) at 5-year follow-up (**f**), and significant correlation with Health Assessment Questionnaire (*HAQ*) score at 6- to 10-year follow-up (95 % CI = 0.14 to 0.90) (**g**). β values indicates the slope for linear regression and *p* value indicates if the slope is significantly non-zero. *p < 0.05; **p < 0.01. *CI* confidence interval, *r*_*s*_ Spearman correlation coefficient

Next, we investigated whether clinical response in patients was associated with the number and phenotype of T-cell subsets in the muscle biopsies. When patients were compared based on the FI improvement (ΔFI), the group with ΔFI ≤20 % contained significantly higher number of both CD3+ cells/mm^2^ (median 254.4 versus 6.1, *p* = 0.004) (Fig. 4a) and CD244+ cells/mm^2^ (median 89.5 versus 2.3, *p* = 0.004) (Fig. 4b) before treatment than the group with ΔFI >20 %. Additionally, patients in the FI-low group post-treatment (Fig. 1b) were found to display higher levels of CD244+ cells (median 13.8 cells/mm^2^, *p* = 0.01) in post-treatment muscle biopsies compared to those in the FI-high group (median 0.8 cell/mm^2^) (Fig. 4c). CD244+ cells/mm^2^ in post-treatment muscle biopsies also displayed an almost significant moderate negative correlation with FI after treatment (r_s_ = –0.53, *p* = 0.07) (Fig. 4d).

CD244+ cells/mm^2^ in post-treatment muscle biopsies were also associated with various clinical parameters at long-term follow-up in patients. Post-treatment CD244+ cells/mm^2^ correlated significantly with patient disease activity MITAX at 5-year follow-up (r_s_ = 0.64, *p* = 0.03) (Fig. 4e), and also displayed negative correlation with muscle strength in patients at 5-year follow-up, measured by MMT8 (r_s_ = –0.66, *p* = 0.05) (Fig. 4f). The patient disability measured by HAQ score showed positive correlation at 5-year follow-up (r_s_ = 0.59, *p* = 0.05, *n* = 11), and at 6- to 10-year follow-up (r_s_ = 0.67, *p* = 0.02) (Fig. 4g). In addition to correlations, CD244+ cells/mm^2^ post-treatment also displayed almost significant linear regression with FI post-treatment (Fig. 4d) and MITAX at 5 years (Fig. 4e) and significant linear regression with MMT8 at 5 years (Fig. 4f).

### CD4+CD28null T-cell subset display resistance to glucocorticoid and Treg-mediated immunosuppression in vitro

We have previously demonstrated that the majority of CD244 expressing T cells display a CD28null phenotype in the circulation of myositis patients. Here, a flow cytometry-based in vitro immunosuppression assay was used to evaluate suppressive effects of glucocorticoids and Tregs on activation-induced CD69 upregulation on the reciprocal CD28null and CD28+ T-cell subsets.

**Fig. 5:**
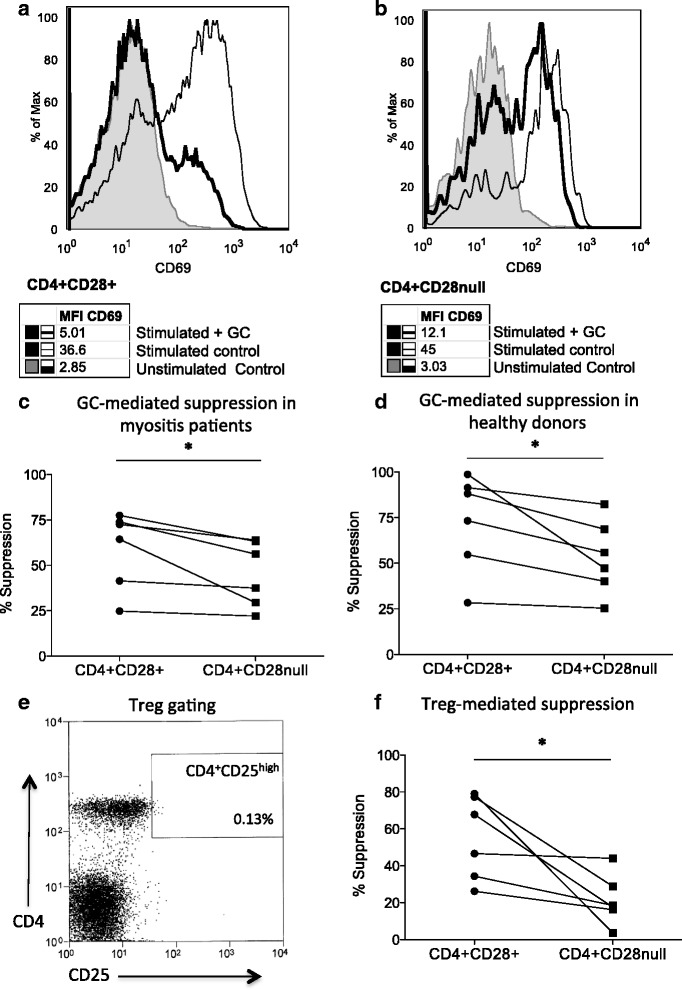
Resistance to glucocorticoid and regulatory T cell (*Treg*)-mediated immunosuppression in circulatory CD4+CD28null T cells. A flow cytometry-based in vitro immunosuppression assay using activation-induced CD69 upregulation was used to evaluate suppressive effects of glucocorticoid (*GC*) and Tregs on T-cell subsets. Representative flow cytometry histograms depicting glucocorticoid-mediated suppression of CD4+CD28+ and CD4+CD28null T cell subsets in myositis patients are shown in (**a**) and (**b**), respectively. *Grey* tinted histograms depict CD69 levels on unstimulated control T cells, *black line* depicts CD69 upregulation on stimulated control T cells and the *bold black line* depicts glucocorticoid-induced suppression of CD69 upregulation. The histograms show lower suppression of CD28null T cells compared to CD28+ T cells. At group level also, CD4+CD28null T cells were more resistant to glucocorticoid-mediated suppression compared to CD28+ both in myositis patients (*n* = 6) (**c**) and in healthy donors (*n* = 6) (**d**). Viable Tregs were isolated based on a CD3+CD4+CD25^high^ gating strategy, as shown in the representative flow cytometry dot plot (**e**). CD4+CD28null T cells were more resistant to Treg-mediated suppression compared with CD28+ T cells (*n* = 6) (**f**). *p < 0.05. *MFI* mean fluorescence intensity

Representative flow cytometry histograms depicting glucocorticoid-mediated suppression of CD4+CD28+ and CD4+CD28null T-cell subsets in myositis patients are shown in Fig. 5a and b, respectively. As shown in the examples and in the summary graph (Fig. 5c), CD4+CD28null T cells were less sensitive towards glucocorticoid-mediated suppression compared to their CD28+ counterparts in myositis patients (median suppression: 46.8 % versus 68.5 %, *n* = 6). Also, in healthy donors, CD4+CD28null T cells displayed a lower sensitivity towards glucocorticoid-mediated suppression than their CD28+ counterparts (median suppression: 51.6 % versus 80.7 %, *n* = 6) (Fig. 5d).

Treg-mediated in vitro immunosuppression assay could only be performed in healthy donors since Tregs are a rare T-cell subset requiring large sample volumes for their isolation. To sort live Tregs, CD3+CD4+CD25^high^ T cells were selected as Tregs (Fig. 5e). In the suppression assay, CD4+CD28null T cells were found to be less sensitive to Treg-mediated suppression compared with their CD28+ counterparts (median suppression: 18 % versus 57.2 %) (Fig. 5f). No clear trend could be observed in CD8+ compartment with regard to either glucocorticoid or Treg immunosuppression (data not shown).

## Discussion

Despite high doses of glucocorticoids and other immunosuppressive drugs, immune infiltrates (with significant proportion of T cells) often persist in the muscle tissue of patients with PM and DM. In this study, we could demonstrate that CD244+ cells (a validated surrogate marker for CD28null T cells in myositis muscle tissue) dominate the T-cell infiltrates over FOXP3+ cells (a marker for regulatory T cells) in inflamed myositis muscle tissue. After treatment, CD244+ cells were found to be unchanged, while the number of FOXP3+ cells declined, leading to a relative increase in the proportion of CD244+ cells over FOXP3+ cells. Interestingly, patients with higher number of CD244+ cells in muscle tissue at baseline displayed poorer clinical improvement. Furthermore, the higher number of CD244+ cells in the post-treatment biopsies also correlated with a poor clinical response, both in the short- and long-term perspective. Using in vitro assays, we could further demonstrate that both glucocorticoids and CD4+CD25^high^ Tregs are less capable of suppressing CD4+CD28null T cells compared to their CD28+ counterparts, emphasizing the treatment-resistant nature of this T-cell phenotype.

CD28null T cells phenotypically belong to a highly differentiated effector memory T-cell subset with oligoclonal expansions and devoted effector functions [14, 42]. A high frequency of CD28null T cells in the circulation and in the inflamed muscle of PM, DM and inclusion body myositis (IBM) has previously been reported from our group [27, 28]. We have demonstrated that a majority of CD28null T cells (89 % of the CD4+CD28null and 98 % of the CD8+CD28null T cells) in the circulation of patients with myositis stained positive for CD244 and strong correlations were observed between CD244+ cells expressing CD3+ and CD28null T-cell subsets [27]. Also, using a triple immunofluorescence technique, CD244+ cells in myositis muscle tissue stained mostly positive for CD3; therefore these cells were considered CD28null T cells and not NK cells [27]. The presence of common dominant TCR Vβ amongst circulating CD28null T cells and also in muscle tissue of inclusion body myositis, as demonstrated previously by our group, further strengthen the dominance of CD28null T cells in myositis muscle tissue [28]. Such oligoclonally expanded CD28null T cells in myositis patients also retain their proinflammatory effector functions and are not functionally exhausted [28]. Hence, CD244 was used as a surrogate marker for CD28null T cells in muscle tissue in the current study, even though CD244 expression could tentatively also be expected on gamma/delta T cells and some CD3+CD8+CD28+ T cells. Our results demonstrate that persistence of CD244+ cells (i.e., mainly CD28null T cells) may have a negative effect on muscle fiber function and muscle performance.

The CD28null T cells can contribute both directly and indirectly towards muscle tissue damage. In comparison with conventional CD28+ T cells, CD28null cells of both CD4 and CD8 lineage are hypersensitive to stimulation and can release large amounts of IFNγ and TNF [14, 28, 43]. Excess amount of these cytokines may impair the muscle repair process by shifting the balance towards M1 macrophages, inducing a state of chronic inflammation [44]. In addition, CD28null T cells of both CD4 and CD8 origin can directly kill muscle cells in their proximity by secreting granzyme B and perforin [45]. Expression of activating NK receptor could lower the T-cell activation threshold, which could further predispose these cells to become autoreactive [15].

The presence of FOXP3+ cells (referred as regulatory T cells) in muscle tissue of untreated myositis patients has also been reported previously [29]. The numbers and frequencies of FOXP3 cells found in our cohort are similar with findings in the report by Waschbisch et al. [29]. However, this is the first study which investigates the frequencies FOXP3+ in relation to the CD244+ cells and how their relative proportion is affected in myositis muscle tissue by immunosuppressive treatment. Similar to our previous study in rheumatoid arthritis, where intra-articular glucocorticoid treatment decreased both the number and the frequency of FOXP3+ Tregs in synovial tissue [36], we observed a decrease in the number of FOXP3+ cells in myositis muscle tissue upon glucocorticoid-based immunosuppresive treatment. This may have functional consequences since FOXP3+ Tregs have recently been demonstrated to contribute to muscle repair and regeneration in mice [25, 26]. Together, the reduced number of FOXP3+ Tregs and the higher number of muscle-infiltrating CD28null T cells could impair the muscle homeostasis and repair process, leading to chronic inflammation and muscle damage.

Glucocorticoids not only interfere with signaling pathways related to immune activation but are also at high doses capable of inducing apoptosis in immune cells, including T cells [46, 47]. Other immunosuppressive drugs commonly used in combination with glucocorticoids such as methotrexate, azathioprine, cyclophosphamide, and cyclosporine block the proliferative properties of T cells and other immune cells [48]. Of note, CD28null T cells are reported to be long-lived, resistant to apoptosis in vivo [9–11], and also display properties of replicative senescence [49–51]. The unusual in vivo survival and resistance to apoptosis in CD28null T cells is mediated by the anti-apoptotic protein Bcl-2 [9] and also by proteasome-mediated reduction in pro-apoptotic molecules such as Bim [12].

Of note, studies in the animal model of experimental autoimmune encephalomyelitis have demonstrated that the density of membrane-bound glucocorticoid receptors (GCRs) on T cells is critical for apoptosis induction, and T cells with a higher density of GCR expression are more susceptible to glucocorticoid-mediated apoptosis compared to those with a lower density of GCR expression [52]. Interestingly, a recent study showed a significant loss of GCR expression in CD28null T cells in patients with chronic obstructive pulmonary disease (COPD) as well as in healthy controls [53]. Hence, it is possible that CD28null T cells can escape the apoptotic effects of high doses of glucocorticoids as well as anti-proliferative effects of concomitant immunosuppressive drugs, leading to their persistence in muscle tissue of patients with myositis.

CD28null T cells proliferate poorly in in-vitro conditions due to their replicating senescent properties [49–51], and we have also demonstrated this in the setting of myositis [27]. Therefore, we chose to study the effect of CD25^high^ Tregs on T cell activation in vitro, instead of previously used approaches to study proliferation [30]. Activation can be measured by upregulation of early T-cell activation markers such as CD40L and CD69. However, CD40L is not adequately expressed on CD28null T cells [54]; therefore, we focused on CD69, after having validated that CD69 upregulation occurs on CD28null T cells in both the CD4+ and CD8+ compartment. In addition to CD4+CD28null T cells being less sensitive to Tregs, we report that CD4+CD28null T cells are also less sensitive to glucocorticoid-mediated suppression in vitro. The reduced sensitivity to suppression in CD28null T cells could be due to distinct signaling mechanism in these cells, e.g., NK-related receptors on CD28null T cells could potentially enhance or modulate their activation and function leading to a different mode of suppression [15].

There are some limitations with the present study. The cohort is small, due to PM/DM being rare disorders and due to the invasive procedure with repeated muscle biopsies. There is also clinical heterogeneity, e.g., one patient had anti-SRP antibodies, although without signs of necrotizing myopathy. One patient also had Sjögren’s syndrome, but her biopsy was compatible with PM and thus her Sjögren was determined as secondary. The group of patients is also heterogenous regarding treatment at time of the second biopsy. In this observational study the patients received similar starting doses of prednisolone combined with a disease-modifying drug, and a repeat muscle biopsy was planned at 6 months follow-up, but due to issues unrelated to the effect of treatment the median duration between start of treatment and repeat biopsy was between 4 and 12 months. It is therefore possible that differences in the total dose of steroids at the time of repeat biopsy may have affected our results; however, there was no correlation between cumulative dose of prednisolone or treatment duration with the level of CD3+ T cells or CD244+ T cells or FOXP3+ T cells in the second biopsy, so we regard this effect as minor.

Furthermore, long-term clinical data were not available for all patients leading to limitations in statistical analyses. To curtail such limitations, we have pooled the data from PM and DM. Although, we are aware of subtype differences, both subtypes have patients with high frequencies of CD244+/CD28null T cells in the circulation and in the muscle tissue, and these facets were important inclusion criteria in our study. The pooling of data may provide insight into common pathogenic mechanisms in different subtypes of disease. Our current study focused on patients with T-cell infiltrates in muscle and elevated frequencies of CD28null T cells in the circulation, a feature not shared by all myositis patients. Nevertheless, inflammation and T cells persist in a group of patients after months of immunosuppressive treatment (Fig. 3a). Therefore, the results in this study are particularly relevant in the context of myositis patients with persistent immune cell infiltrate.

A clinical response following treatment was supported by decreased serum levels of creatine kinase and improved FI score at the group level; however, the functional improvement was partial. Patients with relatively poor response (FI <75 %) had a significantly higher number of CD244+ cells in post-treatment muscle biopsies compared to patients in the FI-high group. A high number of CD244+ cells/area after approximately 8 months with immunosuppressive treatment correlated with both short-term and long term (5–10 years) clinical outcomes. These findings imply a clinical value for a repeated muscle biopsy after 6–8 months of immunosuppressive treatment and that patients with persistent T cell and CD244+ cells in muscle tissue may require novel treatment strategies.

## Conclusions

We have demonstrated that the current treatment regime based on high doses of glucocorticoids in combination with conventional immunosuppressive agents is insufficient to eliminate CD3+ and CD244+ cells (focusing on the subgroup of myositis patients displaying such cells). We found that poor outcome from the immunosuppressive therapy is linked to persistence of CD244+ cells in muscle tissue in a subset of patients. These CD244+ cells are likely CD28null T cells and immunosuppression resistant, especially the CD4+CD28null T cells. Future studies are needed to investigate the precise phenotype of muscle-infiltrating CD244+ cells. Nevertheless, these findings provide mechanistic insight into the inefficacy of the current treatment approach and directions for novel and improved therapies.

## Abbreviations

DM: dermatomyositis
FI: Functional Index
GCR: glucocorticoid receptor
GMFI: geometrical mean fluorescence intensity
HAQ: Health Assessment Questionnaire
IFNγ: interferon gamma
MITAX: Myositis Intention To Treat Activity Index
MMT8: Manual Muscle Testing 8
NK: natural killer
PBMC: peripheral blood mononuclear cell
PM: polymyositis
r_s_: Spearman correlation coefficient
s-CK: serum levels of creatine kinase
STM: stimulated
TNF: tumor necrosis factor
Treg: regulatory T cell
UNSTM: unstimulated
